# 

**Published:** 1887-10

**Authors:** 


OCTOBER, 1887.

### THE AMERICAN JOURNAL OF DENTAL SCIENCE.

PUBLISHED MONTHLY.

$2.50 PER ANNUM,

EDITED BY

F. J. S. GORGAS, M. D., D. D. S.

VOL. 21 THIRD SERIES. NO. 6.

BALTIMORE:

SNOWDEN & COWMAN.

LONDON:

TRUBNER & CO., 60 PATERNOSTER ROW.

PAYABLE IN ADVANCE.

